# (-)-SCR1693 Protects against Memory Impairment and Hippocampal Damage in a Chronic Cerebral Hypoperfusion Rat Model

**DOI:** 10.1038/srep28908

**Published:** 2016-06-28

**Authors:** Xiaoyin Zhu, Jingwei Tian, Songmei Sun, Qiuju Dong, Fangxi Zhang, Xiumei Zhang

**Affiliations:** 1 Department of Pharmacology, Shandong Univeristy School of Medicine 44#, Wenhua Xi Road, Jinan, Shandong, 250012 P.R. China; 2 School of Pharmacy, Key Laboratory of Molecular Pharmacology and Drug Evaluation (Yantai University), Ministry of Education, Collaborative Innovation Center of Advanced Drug Delivery System and Biotech Drugs in Universities of Shandong, Yantai University, Yantai 264005, P.R. China

## Abstract

Chronic cerebral hypoperfusion (CCH) is one of the most common causes of vascular dementia (VaD) and is recognised as an etiological factor in the development of Alzheimer’s disease (AD). CCH can induce severe cognitive deficits, as assessed by the water maze task, along with neuronal loss in the hippocampus. However, there are currently no effective, approved pharmacological treatments available for VaD. In the present study, we created a rat model of CCH using bilateral common carotid artery occlusion and found that (-)-SCR1693, a novel compound, prevented rats from developing memory deficits and neuronal damage in the hippocampus by rectifying cholinergic dysfunction and decreasing the accumulation of the phospho-tau protein. These results strongly suggest that (-)-SCR1693 has therapeutic potential for the treatment of CCH-induced VaD.

Vascular dementia (VaD) is the most common cause of dementia after Alzheimer’s disease (AD)^1^. VaD is defined as a loss of cognitive function resulting from ischaemic, ischaemic-hypoxic or haemorrhagic brain tissue lesions due to cardiovascular disease and cardiovascular pathological changes^2^. Chronic cerebral hypoperfusion (CCH) is a major cause of VaD and can result from disorders that affect the cerebral vascular system, including hypertension, diabetes, generalised atherosclerosis, and cigarette smoking^3^. A study focusing on the pathogenetic mechanism of VaD has revealed that, similar to AD, cholinergic abnormalities are associated with a disturbance in cognitive function in patients with VaD^4^. Cholinergic neurons that project into the hippocampus play a critical role in learning and memory function, and the cholinergic terminals in the presynaptic membrane are sensitive to ischaemic insults^5^. These findings suggest the possibility of using cholinergic substances as therapeutic interventions in patients with VaD. The inhibition of brain acetylcholinesterase (AChE) can increase synaptic concentrations of acetylcholine, which may improve cognitive dysfunction and neuropathology in patients suffering from cerebral ischaemic dementia^6^. However, many clinical studies have revealed that memantine and the AChE inhibitors donepezil, galantamine and rivastigmine only have modest beneficial effects on the cognitive symptoms of VaD and provide no concomitant global or clinical benefits in most cases^7^. The use of AChE inhibitors is limited because of adverse drug reactions, which include increased patient mortality^7^. This limitation indicates that the inhibition of cholinergic abnormalities may not completely prevent the development of VaD.

The bilateral common carotid artery occlusion (BCCAo) rat model is a commonly used model of VaD. Surgical ligation of both the common carotid arteries in rats produces a CCH condition^8^. Previous studies have revealed that CCH induces severe cognitive deficits, as assessed by the water maze task, along with neuronal loss in the hippocampus^9^. In addition to cholinergic abnormalities, CCH treatment induced learning/memory alterations, increased microtubule-associated protein tau hyperphosphorylation, and caused imbalances in the phosphorylation system by activating glycogen synthase kinase 3β (GSK-3β) and Akt^10^. In humans, tau plays a key role in regulating microtubule dynamics, axonal transport and neurite outgrowth, and all of these functions of tau are modulated by site-specific phosphorylation^11^. Hyperphosphorylation of tau can result in the self-assembly of tangles of paired helical filaments and straight filaments, which are involved in the pathogenesis of AD and other tauopathies, such as VaD^12^. After phosphorylation at additional sites, including Ser396/404, tau can be cleaved, which increases the propensity of tau to oligomerise and eventually form filamentous aggregates^13^. The exact function that tau oligomers and filaments serve in the cell dysfunction/death process has not yet been clearly defined. The enzyme GSK-3β is one of a group of proline-directed kinases that can phosphorylate tau. Gene-knockout studies indicate that both tau and GSK-3β bind to the same region of presenilin 1 (PS1), residues 250–298, whereas the binding domain on tau is the microtubule-binding repeat region^14^. The ability of PS1 to bring tau and GSK-3β into close proximity suggests that PS1 may regulate the interaction between tau and GSK-3β^14^. Protein kinase B, also known as Akt, is activated by phosphorylation at serine 473 (Ser473) and threonine 308 (Thr308). Activated Akt phosphorylates a wide range of substrates, resulting in the activation of anti-apoptotic (survival) factors and the inactivation of pro-apoptotic factors^15^. Akt down-regulates the activities of GSK-3β by phosphorylating serine residue 9 (Ser9)^16^.

The novel compound (-)-SCR1693, whose name is gem-dimethyl-tacripyrine hydrochloride and whose chemical name is (-)-ethyl 5-amino-4-(2-chlorophenyl) -2,7,7-trimethyl-1,4,6,7,8,9-hexahydrobenzo[b][1,8]naphthyridine-3-carboxylate hydrochloride, shows multiple activities at the enzymatic and cellular levels, including the inhibition of both tau hyperphosphorylation and AChE activity. (-)-SCR1693 is being developed as a new drug for the treatment of dementia associated with AD, and its efficacy in AD has been demonstrated in animal models.

Two major pathological pathways leading to the development of AD have been hypothesised: the amyloid cascade and vascular injury. The vascular hypothesis suggests that ischaemic changes and hypoperfusion associated with aging, as well as other vascular risk factors, disturb the blood supply and metabolism. This disturbance leads to neuronal and/or neuroglial energy failure that not only causes injury but also accelerates amyloid over-production and reduces clearance^17^, eventually leading to AD pathology. This vascular hypothesis has also been suggested to contribute to VaD pathology. Thus, medications administered for AD are probably effective in the treatment of VaD. (-)-SCR1693 has been proposed to improve cognitive dysfunction and prevent neurodegeneration in VaD patients by inhibiting AChE and increasing synaptic concentrations of acetylcholine, thus inhibiting tau hyperphosphorylation. Based on these properties, a new trial was initiated using BCCAo rats to test the effects of long-term (-)-SCR1693 administration on behaviour and on neuronal survival and tau pathology.

## Results

### (-)-SCR1693 prevents CCH-induced learning and memory deficits

**Figure 1:**
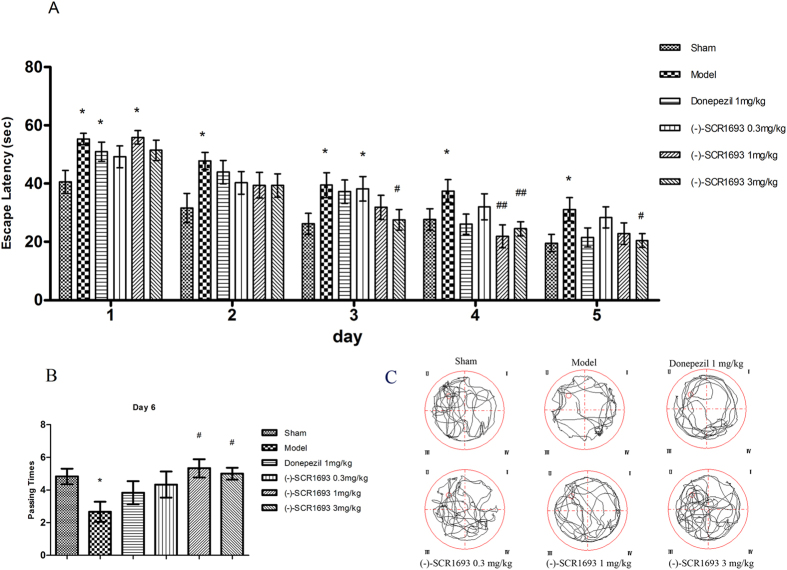
Effects of (-)-SCR1693 on water maze performance deficits caused by permanent bilateral ligation of the common carotid arteries in rats (mean ± SEM, n = 6) . (**A**) In the place navigation test, mean daily escape latencies during the training phase are shown. (**B**) The number of times the rats crossed the place where the platform had been hidden during the training phase of the probe trial (swimming 120 s without platform) is shown. (**C**) Representative swim paths during the probe test are shown. Statistical analysis was performed using one-way ANOVA. When the ANOVA indicated significant treatment effects, the means were separated using Tukey’s multiple comparison test. Bars represent mean ± SEM for replicate samples (n = 6). *p < 0.05, **p < 0.01 vs. Sham group; #p < 0.05, ##p < 0.01 vs. Model group. Representative swim paths during the probe test showed that spatial memory deficits improved in ischaemic rats treated with (-)-SCR1693.

Clinical observations and experimental research have indicated that CCH could lead to learning/memory deficits in subjects who are middle-aged and older^18^. As shown in Fig. 1A, the ischaemic rats in the model group exhibited significantly longer escape latencies than the rats in the sham group from day 1 to day 5 (day 1, p = 0.012; day 2, p = 0.011; day 3, p = 0.019; day 4, p =  0.024; day 5, p = 0.016; n = 6) in the water maze task, which was initiated 4 weeks after BCCAo. On days 2–5, there were no significant differences in escape latencies between the rats in the donepezil and (-)-SCR1693 groups vs. the sham group, except in the 0.3 mg/kg (-)-SCR1693 group on day 3 (p = 0.036). We found that on days 3–5, 3 mg/kg of (-)-SCR1693 significantly shortened the escape latencies prolonged by permanent BCCAo (day 3, p = 0.036; day 4, p = 0.006; day 5, p = 0.026) compared with the model group. On day 4, the escape latencies of the rats in the group that received 1 mg/kg of (-)-SCR1693 were significantly shorter compared with the model group (p = 0.005). Although the mean escape latency of the rats that received 1 mg/kg of donepezil was lower on days 4 and 5, no statistically significant difference was observed compared with the model group during the trial.

In the probe trial, Fig. 1B shows that the number of times the rats crossed the platform area for the experimental and sham groups was 4.83 ± 1.07 and 2.67 ± 1.37 (p = 0.015, n = 6), respectively. Administering 1 mg/kg and 3 mg/kg of (-)-SCR1693 significantly increased the number of times the rats crossed compared with the model group (5.33 ± 1.25 and 5 ± 0.82, p = 0.038 and p = 0.028, respectively), which was similar to the sham group. The number of times the rats crossed the platform area in the group that received 1 mg/kg of donepezil was not different from that in the model group. In Fig. 1C, representative swim paths during the probe test are shown, and they reveal shorter swimming distances and less time spent in the target quadrant for the ischaemic rats, while (-)-SCR1693 ameliorated these changes.

### (-)-SCR1693 alleviates neuronal damage caused by CCH

Global ischaemia is known to often lead to delayed neuronal death in the CA1 region of the hippocampus. The cognitive deficits caused by ischaemia have been shown to be closely correlated with neuronal cell damage in the hippocampal CA1 region^19^. In addition, granule neurons of the dentate gyrus receive inputs from the entorhinal cortex through the perforant pathway and send signals to the CA3 pyramidal neurons through the mossy fibre pathway, which are critical pathways for memory processing and recording. Therefore, neuronal degeneration in these areas, together with synaptic deficits, may underlie the basis of the mild memory impairments observed in CCH mice^20^. Previous studies have shown neuronal damage, such as pyknosis of nuclei and vacuolation of neuropil, in pyramidal neurons of the CA1–CA4 region of the hippocampus and granule neurons of the dentate gyrus (DG) in post-ischaemic rats^19^.

**Figure 2:**
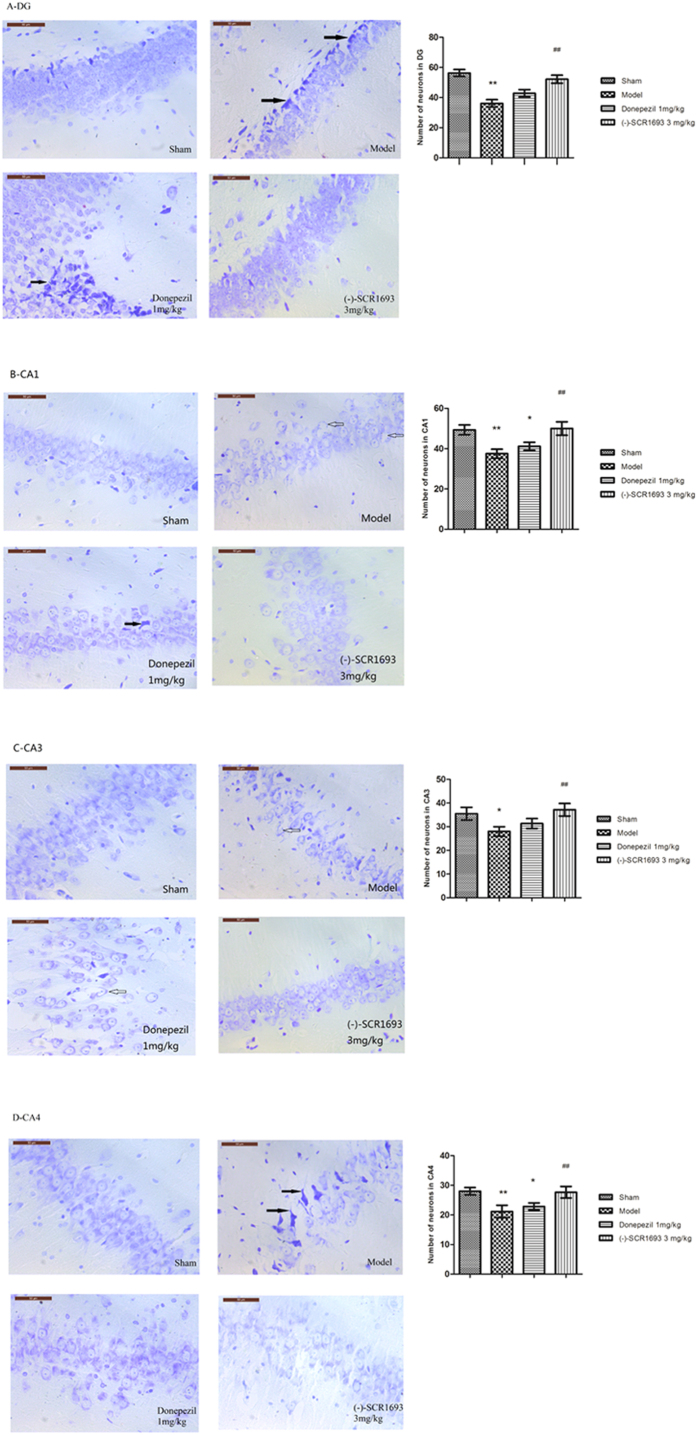
Typical neuropathological changes observed in the hippocampus 4 weeks after bilateral ligation of the common carotid arteries. Neuronal loss, shrinkage (solid arrows) and vacuolation (hollow arrows) of neurons were observed in the CA1, CA3, CA4 and DG regions of the hippocampus in the model group rats. Long-term administration of (-)-SCR1693 and donepezil attenuated chronic hypoperfusion-induced neuronal damage to different degrees (scale bars = 50 μm). Histograms show the neuron number per field of view (×400) in the CA1, CA3, CA4 and DG regions (mean ± SEM, n = 6). *p < 0.05 vs. Sham group; ^#^p < 0.05 vs. Model group. **p < 0.01 vs. Sham group; ^##^p < 0.01 vs. Model group.

The neuroprotective effects of donepezil and (-)-SCR1693 were evaluated by measuring neuronal cell density in the hippocampus after 34 days of global cerebral ischaemia. Representative photomicrographs of the Nissl staining results for each group are shown in Fig. 2. In the sham group, the neurons in the DG, CA1, CA3, and CA4 regions were normal and did not show any cell damage. In ischaemic rats, the numbers of cells in these regions were markedly decreased and the neurons exhibited a shrunken morphology (solid arrows) and vacuolation (hollow arrows). These results demonstrate that ischaemia leads to obvious neuron loss, disordered arrangement, vacuolation of the cell body of neurons, pyknosis of nuclei, and coagulation necrosis in the DG, CA1, CA3, and CA4 regions of the model group. Similar changes were observed in the donepezil group, but these changes were ameliorated in the group that received 3 mg/kg of (-)-SCR1693. In 2 out of 6 rats in the 0.3 mg/kg group and 3 out of 6 rats in the 1 mg/kg group, the histological lesions in the hippocampus were attenuated, but there were no statistically significant differences in the number of surviving neurons in the groups that received 0.3 or 1 mg/kg of (-)-SCR1693 compared with the model group. The histograms show that the number of surviving neurons in the model group was markedly decreased compared with that in the sham rats. Obvious recovery of neuron loss was observed in rats treated with 3 mg/kg of (-)-SCR1693, while the neuron loss could not be suppressed following treatment with 1 mg/kg of donepezil.

### (-)-SCR1693 inhibits AChE activity in the hippocampus and blood plasma

**Figure 3:**
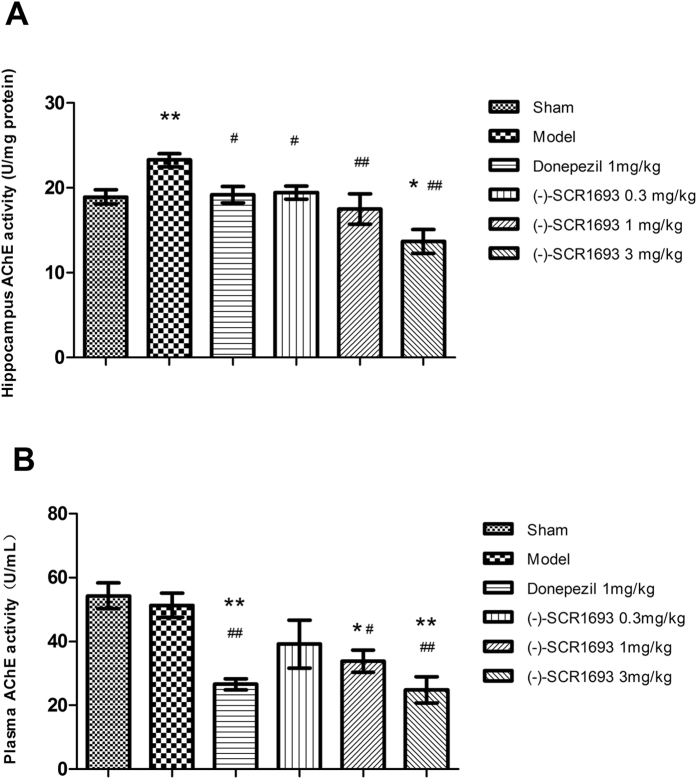
The effects of (-)-SCR1693 on acetylcholinesterase (AChE) activity in the hippocampus and blood plasma (mean ± SEM, n = 6). (**A**) Hippocampal AChE activity. (**B**) Blood plasma AChE activity. *p < 0.05 vs. Sham group; ^#^p < 0.05 vs. Model group. **p < 0.01 vs. Sham group; ^##^p < 0.01 vs. Model group.

Inhibition of AChE activity could decrease the hydrolysation of Ach and increase the concentration of ACh. In Fig. 3a, we show that CCH significantly increased the activity of AChE in the rat hippocampus compared with the sham group (p = 0.002, n = 6). Hippocampal AChE activity in the donepezil group was significantly lower than in the model group (p = 0.018). All three doses of (-)-SCR1693 significantly decreased the activity of AChE compared with the model group (0.3 mg/kg, p = 0.025; 1 mg/kg, p = 0.001; 3 mg/kg, p = 0.000).

As shown in Fig. 3b, blood plasma AChE activity was slightly lower in the model group than in the sham group, but the difference was not statistically significant (p = 0.636). Donepezil (p = 0.001 vs. sham, p = 0.003 vs. model), 1 mg/kg of (-)-SCR1693 (p = 0.013 vs. sham, p = 0.038 vs. model), and 3 mg/kg of (-)-SCR1693 (p = 0.000 vs. sham, p = 0.001 vs. model) significantly decreased blood plasma AChE activity compared with the sham and model groups. (-)-SCR1693 (1 mg/kg) led to a similar decrease in hippocampal AChE activity and higher plasma AChE activity compared with donepezil, while 3 mg/kg of (-)-SCR1693 led to a similar change in plasma AChE activity and lower hippocampal AChE activity compared with donepezil. According to the data, (-)-SCR1693 may produce fewer side effects and may be more efficient in inhibiting AChE activity than donepezil.

### (-)-SCR1693 up-regulates the expression of brain-derived neurotrophic factor (BDNF) in the hippocampus

**Figure 4:**
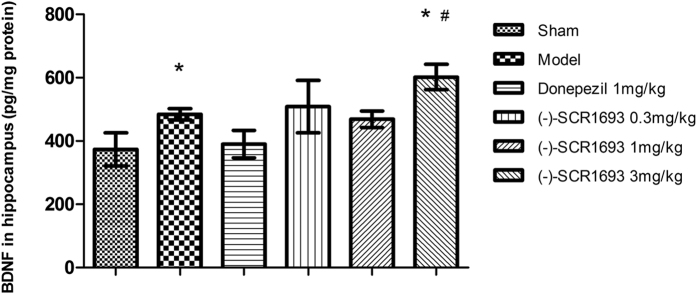
The effects of (-)-SCR1693 on hippocampal brain-derived neurotrophic factor (BDNF) expression (mean ± SEM, n = 6). *p < 0.05 vs. Sham group; ^#^p < 0.05 vs. Model group. **p < 0.01 vs. Sham group; ##p < 0.01 vs. Model group.

The enzyme-linked immunosorbent assay (ELISA) (Fig. 4) revealed that hippocampal BDNF levels in the model group were significantly higher than in the sham group (p = 0.049, n = 6). These results indicate that ischaemia leads to an increase in BDNF gene expression, which is in accordance with a previous study^21^. Exposure to 3 mg/kg of (-)-SCR1693 significantly elevated hippocampal BDNF expression (p = 0.013, vs. sham group; p = 0.045, vs. model group).

### (-)-SCR1693 modulates the phosphorylation of AKT, GSK-3β and tau in the rat hippocampus

**Figure 5:**
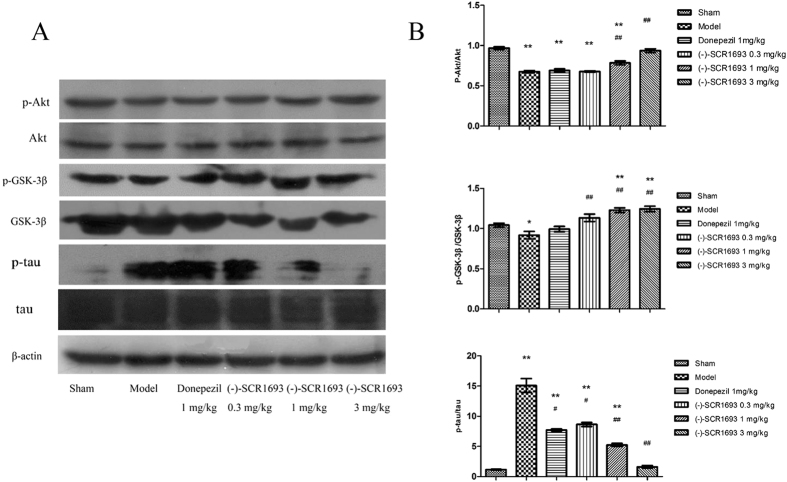
The effects of (-)-SCR1693 on the ratios of p-Akt/Akt, p-GSK-3β/GSK-3β and p-tau/tau (mean ± SEM, n = 6). The full-length blots are shown in the Supplementary Materials. The gels were run under the same experimental conditions. *p < 0.05 vs. Sham group; ^#^p < 0.05 vs. Model group. **p < 0.01 vs. Sham group; ^##^p < 0.01 vs. Model group.

To explore the mechanisms underlying altered tau phosphorylation, we examined the alterations of total Akt, p-Akt, total GSK-3β, and p-GSK-3β. Phosphorylation of Akt at Ser473 reflects an increased activity of Akt^22^. Akt phosphorylation could lead to a decreased activity of GSK-3β, thus reducing tau phosphorylation and activating the cell survival-supporting functions of the Akt pathway^22^. Western blotting (Fig. 5) showed that the levels of total Akt did not change dramatically, while p-Akt immunoreactivity in the hippocampus after CCH treatment decreased by 16%. (-)-SCR1693 (3 mg/kg) reversed this change. The ratio of p-GSK-3β to GSK-3β decreased by 12% following CCH and increased by approximately 10% in rats that were treated with (-)-SCR1693 compared with control rats. Total GSK-3β and p-GSK-3β levels did not change after CCH or after treatment with (-)-SCR1693 and donepezil.

Tau can be phosphorylated at multiple sites, such as Ser396/404, which increases the propensity of tau to oligomerise and eventually form filamentous aggregates^13^. While the protein levels of total tau in all of the groups were similar, a dramatic increase in the levels of p-tau (phosphorylated at Ser396) was observed in the hippocampus of the BCCAo rats. (-)-SCR1693 rectified this CCH-induced change in p-tau, and this effect was dose dependent (Fig. 5). However, there were no obvious changes in the levels of p-Akt and p-GSK-3β between the ischaemic group and either the control group or the (-)-SCR1693 group. We consider the small variations in p-Akt and p-GSK-3β to be unrelated to the dramatic (-)-SCR1693-induced decrease in the levels of p-tau.

### The enzymatic activity of GSK-3β *in vitro*

To determine the effects of (-)-SCR1693 on the activity of the upstream enzymes of tau, an additional cellular functional assay was conducted by Cerep (Le Bois l’Eveque, France). The effects of the compounds on the activity of human CDK5/p35, GSK-3β, MARK1, MARK2, MARK3 and MARK4 were evaluated by measuring the phosphorylation of the corresponding substrates using human recombinant enzymes and the LANCE^®^ detection method. The results shown in Table 1 demonstrate that (-)-SCR1693 did not affect the activities of human CDK5/p35, GSK-3β, MARK1, MARK2, MARK3 and MARK4 *in vitro*.

### The inhibitory effect of (-)-SCR1693 on tau hyperphosphorylation depends on the interaction of PS1 with tau

**Figure 6:**
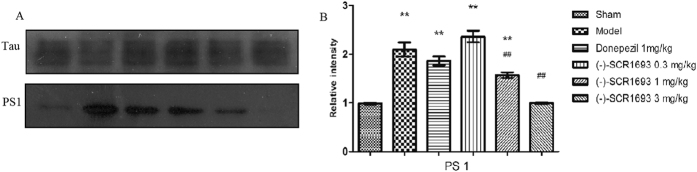
Binding of PS1 to tau using co-immunoprecipitation. (**A**) Protein bands represent the levels of PS1 binding to tau. (**B**) The effects of (-)-SCR1693 on the interaction between tau and PS1 (mean ± SEM, n = 6). The full-length blots are shown in the Supplementary Materials. *p < 0.05 vs. Sham group; ^#^p < 0.05 vs. Model group. **p < 0.01 vs. Sham group; ^##^p < 0.01 vs. Model group.

The ability of PS1 to bring tau and GSK-3β into close proximity suggests that PS1 may play an important role in regulating the phosphorylation of tau via GSK-3β^14^. In the present study, (-)-SCR1693 did not alter either the phosphorylation or enzymatic activity of GSK-3β. Thus, we speculated that the interaction between PS1 and tau may explain the effect of (-)-SCR1693 on tau. The anti-tau antibody was incubated with protein extracts from the hippocampus. The immunocomplexes were then precipitated by incubation with Protein A+G Agarose and detected using the anti-PS1 antibody. As shown in Fig. 6, more PS1 was precipitated in the ischaemia model group than in the control group, which indicates that CCH results in an increase in PS1 binding to tau. (-)-SCR1693 at doses of 1 mg/kg and 3 mg/kg prevented the binding of tau to PS1, but donepezil did not have this effect.

## Discussion

In the present study, we investigated the mechanisms underlying CCH-induced spatial learning and memory deficits, and we examined whether the novel compound (-)-SCR1693 could ameliorate these deficits in a CCH rat model. We also compared the effects of (-)-SCR1693 with the effects of donepezil, which has been shown to improve hippocampal neuronal damage and reduce learning and memory deficits in the hippocampus of a global ischaemic gerbil model^23^. Our findings suggest that (-)-SCR1693 exhibits beneficial effects on CCH-induced cognitive and memory deficits and that its effects on these cognitive deficits are mediated by attenuating CCH-induced dysfunction of the central cholinergic system and the tau protein in the brain.

Surgical ligation of both of the common carotid arteries in rats produces a chronic, global hypoperfusion state, which is less severe than the 4-vessel occlusion (4-VO) animal model^24^. Impaired learning and memory have been confirmed using the Morris water maze task at ~7 days post-surgery^25^. In our present behavioural experiment, learning performance in the water maze task was severely impaired in the BCCAo rats, as indicated by an increase in the time required to find the hidden platform and a decrease in the number of times the rats crossed the place where the hidden platform was previously located. These results are in agreement with previous studies showing that chronic cerebral hypoperfusion results in impaired learning performance^26^. Our behavioural experiment shows that (-)-SCR1693 prevents learning and memory deficits and is more effective than donepezil.

Neurons have a high demand for oxygen and a limited endogenous reserve of glycogen. Thus, adult neurons are vulnerable to ischaemic conditions^27^. Hippocampal neurons are particularly sensitive and undergo selective and delayed degeneration in response to global ischaemia^28^. The pyramidal neurons in the hippocampus are critically involved in spatial learning and memory, and degeneration of these neurons results in cognitive deficits^29^. We found that CCH caused hippocampal neural damage, including neuronal loss, disordered arrangement, pyknosis of nuclei, vacuolation of the neuronal cell body, and coagulation necrosis in the model group. (-)-SCR1693 significantly attenuated the bilateral ligation-induced histological lesions in the hippocampus, while vacuolation and pyknosis of neurons were still observed in the donepezil group. Thus, the neuroprotective effects of (-)-SCR1693 contribute to cognitive rehabilitation, and its efficacy is greater than that of donepezil.

Cholinergic deficits in VaD have been observed in preclinical and clinical studies^30^,^31^, and evidence of cholinergic changes in animal models of VaD has recently been reviewed^32^. The VaD-induced increase in AChE activity in the hippocampal areas could lead to reductions in the efficiency of cholinergic neurotransmission, such as a decrease in ACh levels in the synaptic cleft. This effect would contribute to the progression of cognitive impairment and other types of neurological dysfunction seen in patients and rats with VaD^33^. Our data showed that long-term administration of (-)-SCR1693, a potent AChE inhibitor, significantly inhibited AChE activity in a dose-dependent manner. The inhibition of serum AChE activity by donepezil has been linked to its adverse side effects, which include nausea, vomiting, diarrhoea, abdominal pain, weight loss, anorexia^34^ and hepatotoxicity associated with serum alanine aminotransferase elevation in up to 50% of patients^35^. We could reasonably conclude that (-)-SCR1693 might have similar AChE-inhibiting effects in the hippocampus, but with less adverse side effects, as higher doses of (-)-SCR1693 did not cause more adverse side effects than donepezil. A previous study reported that BDNF is involved in adult hippocampal neurogenesis and memory recovery under ischaemic conditions^36^ and that BDNF expression may be involved in the CREB-dependent neuroprotective mechanisms of donepezil in ischaemic injuries^23^. Neurodegenerative disorders may indeed affect neurotrophic factor function by reducing the adaptation of neurons to disease-related alterations^37^. Our results indicate that CCH induces an increase in BDNF expression, which was previously considered to be induced by neurogenesis after ischaemia^38^. (-)-SCR1693 also increased the levels of BDNF, which may contribute to the protection of neurons in the hippocampus.

To investigate the possible molecular mechanisms underlying CCH-induced cognitive impairment and the effects of (-)-SCR1693, we further investigated tau-related molecules and pathways in the CCH rat brain. Under pathological conditions in which there is an imbalance in the phosphorylation/dephosphorylation of tau, aberrant tau phosphorylation at Ser396 can increase the propensity of tau to oligomerise and eventually form filamentous aggregates^13^. The present study found that the levels of p-tau increased significantly in the hippocampi of the BCCAo rats, while (-)-SCR1693 inhibited the CCH-induced phosphorylation of tau at Ser396. Active GSK-3β can cause hyperphosphorylation of the tau protein at Ser396^11^, and its upstream enzyme, activated Akt, increases Aβ protease levels. This increase inhibits GSK-3β and activates multiple survival signals to promote neuron growth and survival^39^,^40^. The present study found that CCH led to a minor decrease in p-Akt (active form) and p-GSK-3β (inactive form), while (-)-SCR1693 increased p-Akt and p-GSK-3β expression to almost normal levels. The effects of (-)-SCR1693 on the phosphorylation of Akt and GSK-3β in the rat hippocampus are clearly not the main cause of the inhibition of tau hyperphosphorylation. In addition, cellular functional assays conducted by CEREP confirmed that (-)-SCR1693 did not affect the enzymatic activity of GSK-3β. Therefore, (-)-SCR1693 inhibited the hyperphosphorylation of tau at Ser396 without affecting the enzymatic activity of GSK-3β. PS1 has been proven to bring tau and GSK-3β into close proximity to regulate the phosphorylation of tau by GSK-3β^14^. In our co-immunoprecipitation study, CCH resulted in more interactions between tau and PS1, which may be the reason why the levels of p-tau increased without changes in p-GSK-3β levels. (-)-SCR1693 decreased the precipitation of PS1, which suggests that (-)-SCR1693 may inhibit the interactions between tau and PS1. Therefore, the phosphorylation of tau by GSK-3β was inhibited. Donepezil did not show similar effects on PS1 and the tau protein. However, further research is needed to investigate the precise mechanism of action of (-)-SCR1693.

In summary, the present study shows that (-)-SCR1693 attenuates the learning/memory impairment that is due to CCH-induced neuron damage. The proposed mechanisms of the neuroprotective effects of (-)-SCR1693 include the rectification of cholinergic deficits, an increase in survival signals such as BDNF, and a decrease in the accumulation of the phospho-tau protein. Our results strongly suggest that (-)-SCR1693 has therapeutic potential for the treatment of VaD-induced cholinergic dysfunction and CCH and shows more therapeutic potential than donepezil. The pathological changes that occur after permanent bilateral ligation of the common carotid arteries are quite similar to those observed in patients suffering from multi-infarct dementia, Binswanger’s and AD^41^. Therefore, the present findings suggest that (-)-SCR1693 may be a promising therapeutic agent for the treatment of other diseases involving vascular dementia. However, further studies are needed to explore its precise mechanism of action.

## Materials and Methods

### Treatment and control materials

(-)-SCR1693 (light yellow solid, purity = 98%, provided by Jiangsu Simovay Pharmaceutical Co., Ltd) was suspended in 1% (w/v) carboxymethyl cellulose sodium (SCMC) solution. Donepezil (Donepezil Hydrochloride Tablets, Eisai, China), which was used as a positive control, was pulverised and suspended in 1% (w/v) SCMC solution. Rats in the sham group were given 1% (w/v) SCMC solution.

### Rat surgery and treatment

Male Sprague–Dawley (SD) rats weighing between 200 and 250 g were purchased from Shandong Luye Pharmaceutical Co. Ltd, China. Animals were housed at 50–70% humidity, a temperature of 22–24 °C, and under a 12:12 h light/dark cycle. All experiments were conducted in accordance with the guidelines of the Ministry of Health of PR China and the Animal Care Committee of China Medical University. The study protocol was approved by the Experimental Animal Research Committee of Yantai University. Food and water were freely available during all phases of the experiment. Rats were acclimated to the facility for 5 days prior to surgery. The rats were anaesthetised with 3 mL/kg of 10% (w/v) chloral hydrate administered by intraperitoneal injection. Through a midline cervical incision, the bilateral common carotid arteries were exposed and gently separated from the carotid sheath and vagus nerve. Each artery of the rats assigned to the ischaemic group was ligated with a 5–0 silk suture, while rats in the sham group underwent the same operation, including the neck incision and isolation of arteries, but without ligation. During recovery, the rats were kept in the animal quarters with free access to food and water. After the operation, the ischaemic animals were randomly divided into 6 groups (n = 12): a sham group, a model group, a 1 mg/kg/day donepezil group, and (-)-SCR1693 groups consisting of 0.3, 1, or 3 mg/kg/day. The SCMC or drugs were administered once daily for 4 weeks by gavage at a dosing volume of 1 mL/kg. Four weeks later, the spatial memory retention of the rats was tested, after which the rats were sacrificed to conduct histopathologic and biochemical examinations. During the behavioural test, the drug was administered 60 min before the trials.

### Morris water maze

Spatial memory performance was evaluated 4 weeks after BCCAo or sham surgery using the Morris water maze^29^. The Morris water maze device is composed of a circular pool placed in a room with conspicuous symbols on the pool wall. The pool was 150 cm in diameter, 50 cm deep, and filled to a height of 30 cm with water to cover a platform (10 cm in diameter). The platform and the interior of the device were painted black to prevent visibility of the platform, which was submerged approximately 1.5 cm below the surface of the water and remained in the middle of quadrant II. The swimming activity of each rat was monitored by a video camera linked to a computer with an image analyser. On days 1–5, the rats were placed in the water (24 ± 1 °C) with their backs to the platform and allowed to swim until they found the platform (maximum swim time 60 s). The latency of each rat climbing onto the hidden platform was recorded. If the animal failed to locate the platform in 60 s, it was placed on the platform and left there for 20 s and the result was recorded as 60 s. Each rat was placed into the water at the same point in one quadrant and then the three other quadrants. On day 6, the platform was removed from the pool and the number of times rats crossed the place where the hidden platform had been previously hidden in 120 s was recorded.

### Histopathological examination

After behavioural examination, six rats from each group were anaesthetised with 3 mL/kg of 10% (w/v) chloral hydrate administered by intraperitoneal injection. After myocardial perfusion with 4% (w/v) paraformaldehyde solution, the brains of the 6 rats in each group were separated and fixed in paraformaldehyde solution overnight. The brains were then embedded in paraffin and cut into sections (5 μm) using a microtome (Leica, Nussloch, Germany; S100, TPI). The tissue slides were stained with Nissl staining solution. Neuronal damage was examined and evaluated by counting the number of surviving neurons per field of view (×400) in the DG, CA1, CA3, and CA4 regions using light microscopy (Leica, DMIRB).

### Measurement of AChE activity

The remaining rats were anaesthetised with 3 mL/kg of 10% (w/v) chloral hydrate administered by intraperitoneal injection, and blood samples were obtained from the abdominal aorta. After myocardial perfusion with 0.9% (w/v) sodium chloride solution, the skull was dissected and the hippocampus was separated on ice. The tissue was homogenised in ice-cold saline to determine the relative biochemical index. AChE activity in blood plasma and the hippocampus was assayed using an AChE assay kit (#A024, Nanjing Jiancheng Bioengineering Institute, China) via a spectrometric method^42^.

### Measurement of BDNF

BDNF expression in the hippocampus was measured using the BDNF E_max_^®^ ImmunoAssay System (#G7610, Promega Corporation, USA) as per the manufacturer’s instructions.

### Western blot analysis

Protein samples were homogenised in lysis buffer (20 mM Tris, 150 mM NaCl, 1% (v/v) Triton X-100) containing 1 mM PMSF, sodium pyrophosphate, β-glycerophosphate, EDTA, Na_3_VO_4_, and leupeptin. The samples were then incubated for 30 min at 4 °C and centrifuged for 10 min at 13,000 × g. The supernatant (lysate) was collected, and 15 μg protein was loaded into each lane. Samples were separated on 8–12% SDS-polyacrylamide gels and electro-blotted onto nitrocellulose membranes (Millipore). After blocking with 5% (w/v) non-fat milk, the blots were incubated with the following primary antibodies: rabbit anti-phospho-Akt (p-Akt-Ser473, 1:400, Santa Cruz), rabbit anti-Akt (1:400, Santa Cruz), rabbit anti-phospho-GSK-3β (p-GSK-3β-Ser9, 1:1000, Cell Signaling Technology), rabbit anti-GSK-3β (1:1000, Cell Signaling Technology), mouse anti-phospho-tau (p-tau-Ser396, 1:1000, Cell Signaling Technology), mouse anti-tau (1:1000, Cell Signaling Technology), rabbit anti-presenilin 1 (1:1000, Cell Signaling Technology), and β-actin (1:1000, Santa Cruz). Conjugated goat anti-rabbit or goat anti-mouse IgG was detected with enhanced chemiluminescence (ECL) (Pierce^®^ ECL Western Blotting Substrate). β-actin was used as an internal reference for relative quantification.

### Cellular functional assays *in vivo*

The effects of (-)-SCR1693 on the activities of human CDK5/p35, GSK3β, MARK1, MARK2, MARK3 and MARK4 were evaluated by measuring the phosphorylation of the respective corresponding substrates using a human recombinant enzyme and the LANCE^®^ detection method (Le Bois l’Evêque - B.P. 1 - 86600 Celle-L’Evescault - FRANCE).

### Co-immunoprecipitation

Protein extracts from the rat hippocampi of each group were preincubated with 1 μg of anti-tau antibody at 4 °C overnight and then incubated with 40 μL Protein A+G Agarose at 4 °C for 3 h. The mixtures were centrifuged at 2500 rpm for 5 min, and then the supernatant was discarded. After being washed 5 times in phosphate buffered saline, the immunoprecipitates were eluted by incubation with loading buffer at 100 °C for 5 min. The immunoprecipitates were subjected to SDS-polyacrylamide gel electrophoresis for detection of tau and PS1.

### Statistical analysis

Statistical analysis of the data was conducted according to the Curran-Everett and Benos methods. All data were expressed as the mean ± SEM, n = 6. The behavioural data were analysed using one-way repeated-measures ANOVA or two-way repeated-measures ANOVA followed by the Tukey test for multiple comparisons among different groups. Neurochemical data were compared using one-way ANOVA followed by the Tukey test for multiple comparisons. P < 0.05 was considered statistically significant.

## Additional Information

**How to cite this article**: Zhu, X. *et al*. (-)-SCR1693 Protects against Memory Impairment and Hippocampal Damage in a Chronic Cerebral Hypoperfusion Rat Model. *Sci. Rep*. **6**, 28908; doi: 10.1038/srep28908 (2016).

## Supplementary Material

Supplementary Information

## Figures and Tables

**Table 1:** Enzyme and cell-based assays profile of (-)-SCR1693.

Assay	Test Concentration (M)	% Inhibition of Control Values	% of Control Values			Reference Compound	IC50 Ref (M)	nH Ref
			1st	2nd	Mean			
CDK5 /p35	3.0E-05	−5	105.2	104.1	104.6	staurosporine	2.1E-08	1.2
GSK3beta	3.0E-05	−3	106.4	98.8	102.6	staurosporine	9.9E-08	1.6
MARK1	3.0E-05	−4	100.7	107.8	104.2	staurosporine	1.5E-08	2.9
MARK2	3.0E-05	7	93.5	91.5	92.5	H-89	8.3E-06	2.9
MARK3	3.0E-05	4	95.8	96.9	96.3	staurosporine	1.7E-08	1.6
MARK4	3.0E-05	2	98.5	97.1	97.8	hymenialdisine	3.6E-08	1.4
