# Supplementary material for: (-)-SCR1693 Protects against Memory Impairment and Hippocampal Damage in a Chronic Cerebral Hypoperfusion Rat Model
Source: Sci Rep. 2016 Jun 28;6:28908. doi: 10.1038/srep28908 (PMC4924100; doi:10.1038/srep28908)
Supplement: Supplementary Information [file srep28908-s1.pdf]

(-)-SCR1693 Protects against Memory Impairment and Hippocampal Damage in a Chronic Cerebral Hypoperfusion Rat Model

Xiaoyin Zhu[a,1,2], Jingwei Tian[2], Songmei Sun[2], Dong Qiuju[2], Zhang Fangxi[2], Xiumei Zhang[b,1] *

[1] Department of Pharmacology, Shandong Univeristy School of Medicine 44#, Wenhua Xi Road, Jinan, Shandong, 250012 P.R. China

[2] Key Laboratory of Molecular Pharmacology and Drug Evaluation (Ministry of Education of China), School of Pharmacy, Yantai University, Yantai, China.

[a]E-mail: zhuxiaoyin@luye.cn

[b]E-mail: zxy_sptenic@163.com

p-Akt

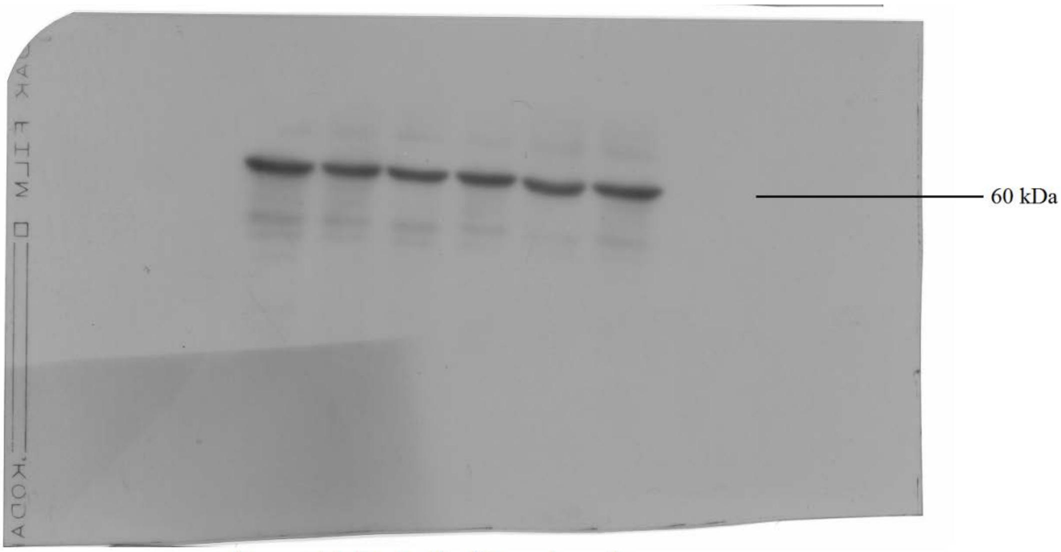

60 kDa

sham model donepezil 0.3 1 3
1 mg/kg (-)-SCR1693 (mg/kg)

Akt

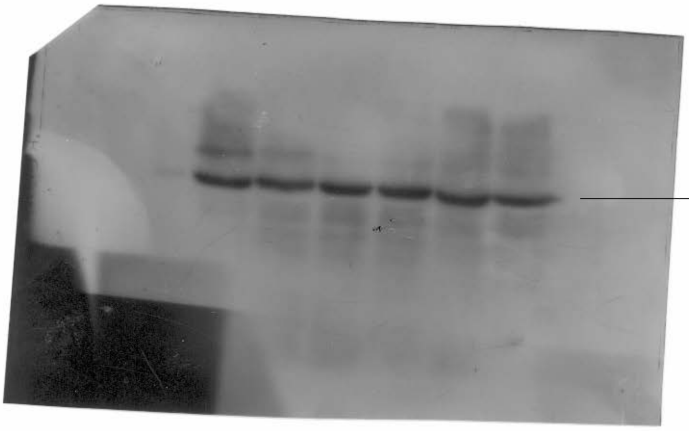

60 kDa

sham model donepezil 0.3 1 3
1 mg/kg (-)-SCR1693(mg/kg)

p-GSK-3β

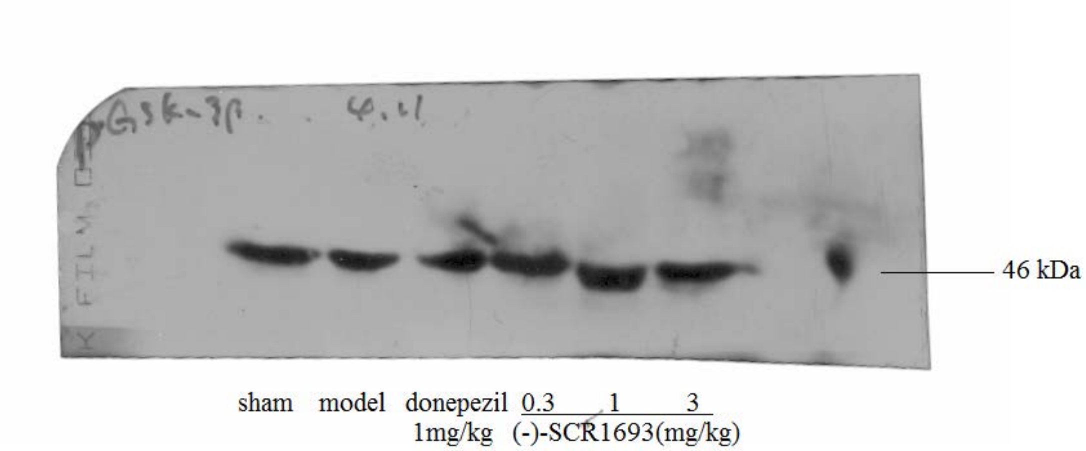

GSK-3β

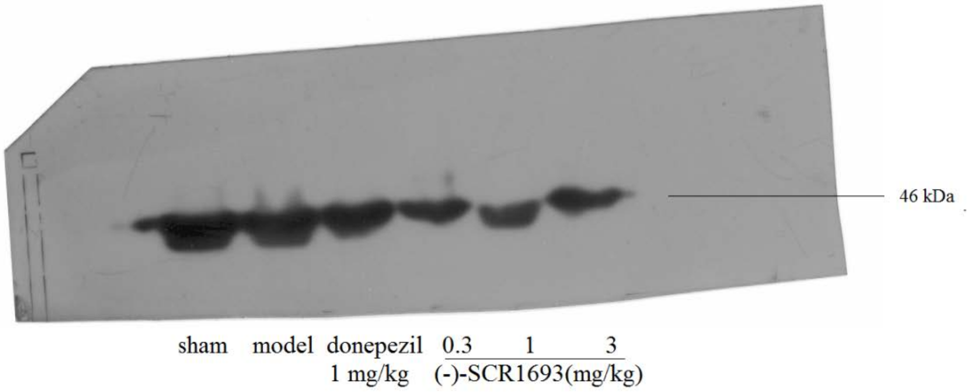

p-tau

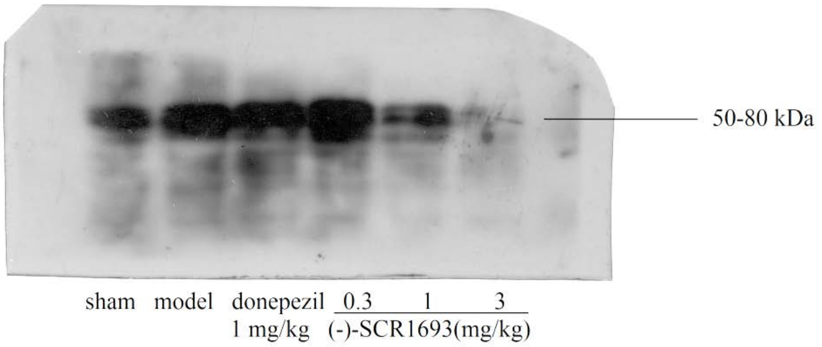
50-80 kDa
sham
model
donepezil
1 mg/kg
0.3
1
3
(-)-SCR1693(mg/kg)

tau

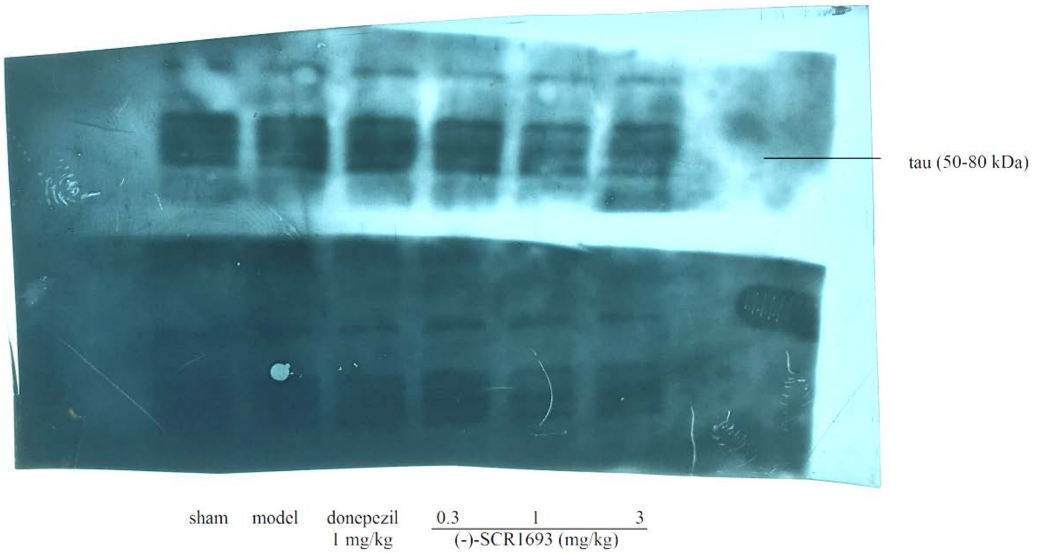
tau (50-80 kDa)
sham
model
donepezil
1 mg/kg
0.3
1
3
(-)-SCR1693 (mg/kg)

β-actin

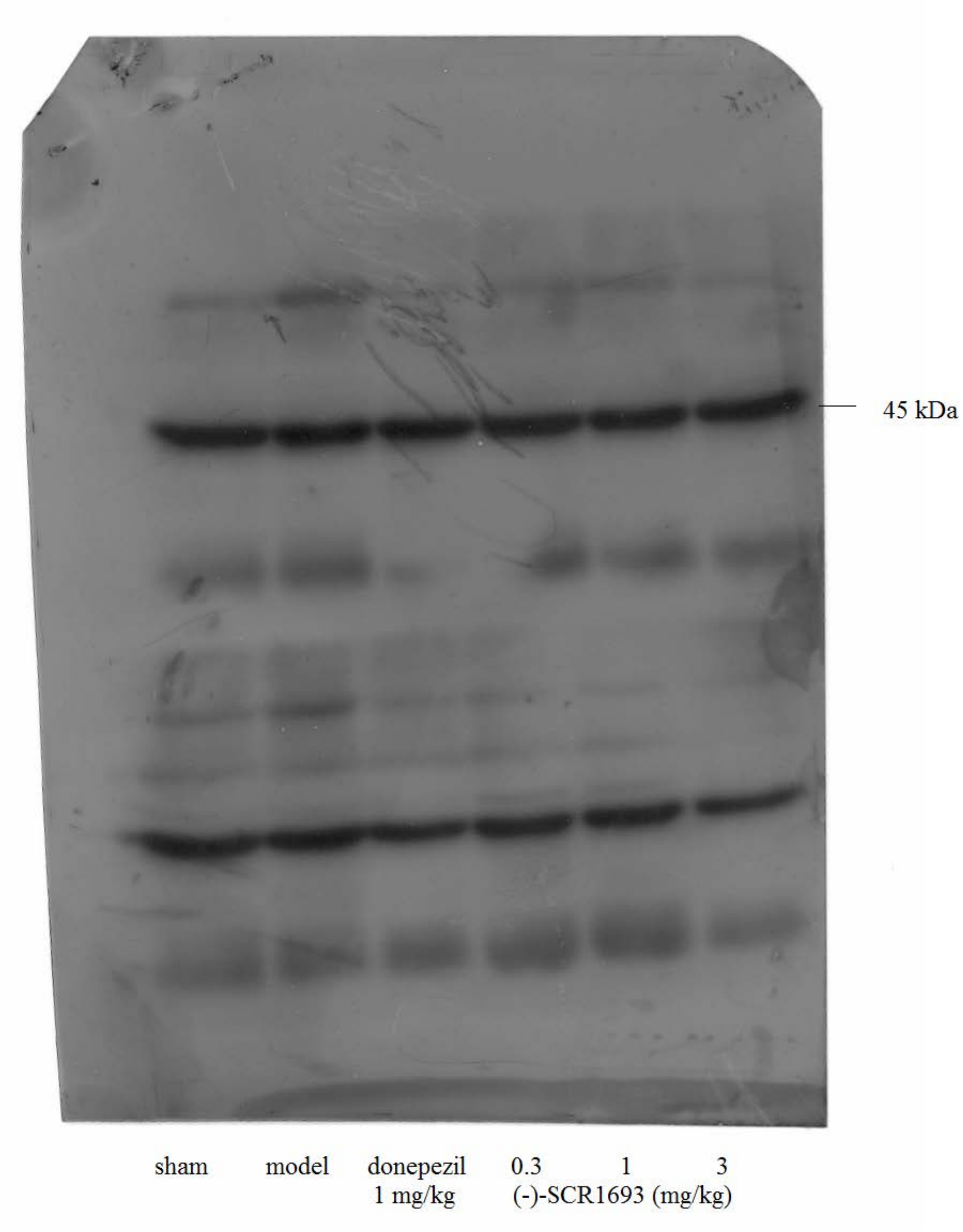

Tau

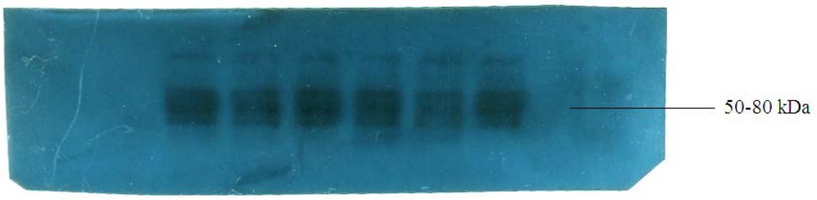

sham model donepezil 0.3 1 3

1 mg/kg (-)-SCR1693(mg/kg)

Presenilin 1

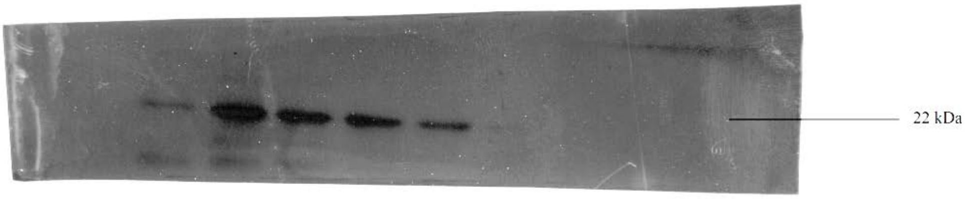

sham model donepezil 0.3 1 3

1 mg/kg (-)-SCR1693(mg/kg)
